# Associations Between Birth Characteristics, Weaning Practices, and the Metabolic Syndrome in Children: A Descriptive Study

**DOI:** 10.3390/metabo15030148

**Published:** 2025-02-22

**Authors:** Teofana Otilia Bizerea-Moga, Tudor Voicu Moga, Ramona Stroescu, Lazar Chisavu, Otilia Mărginean, Flavia Chisavu

**Affiliations:** 1 Department XI of Pediatrics-1st Pediatric Discipline, Center for Research on Growth and Developmental Disorders in Children, ‘Victor Babeș’ University of Medicine and Pharmacy Timișoara, Eftimie Murgu Sq No. 2, 300041 Timișoara, Romania; bizerea.teofana@umft.ro (T.O.B.-M.); stroescu.ramona@umft.ro (R.S.); marginean.otilia@umft.ro (O.M.); 2 1st Pediatric Clinic from ‘Louis Țurcanu’ Children’s Clinical and Emergency Hospital, Iosif Nemoianu 2, 300011 Timișoara, Romania; 3 Department VII of Internal Medicine-Gastroenterology Discipline, Advanced Regional Research Center in Gastroenterology and Hepatology, ‘Victor Babeș’ University of Medicine and Pharmacy Timișoara, Eftimie Murgu Sq No. 2, 300041 Timișoara, Romania; moga.tudor@umft.ro; 4 Gastroenterology and Hepatology Clinic from ‘Pius Brînzeu’ County Emergency Clinical Hospital, Liviu Rebreanu 156, 300723 Timișoara, Romania; 5 4th Pediatric Clinic from ‘Louis Țurcanu’ Children’s Clinical and Emergency Hospital, Iosif Nemoianu 2, 300011 Timișoara, Romania; farkas.flavia@umft.ro; 6 Centre for Molecular Research in Nephrology and Vascular Disease from “Victor Babes” University of Medicine and Pharmacy-Faculty of Medicine, Eftimie Murgu Square No. 2, 300041 Timisoara, Romania; 7 Nephrology Discipline, “Victor Babes” University of Medicine and Pharmacy, Eftimie Murgu Square No. 2, 300041 Timisoara, Romania

**Keywords:** obesity, children, metabolic syndrome, small gestational age

## Abstract

**Background:** Childhood obesity has seen an important rise in recent decades, in both the pediatric and adult populations. Excess weight can cause various health complications, such as the metabolic syndrome (MetS), a cluster of medical conditions linked to adverse cardiometabolic outcomes. Although MetS may be attributed mainly to adults, early life factors, such as birth characteristics and feeding practices, may influence its development in obese children. **Aim:** This study aims to investigate the relationships between birth metrics, early feeding practices, and the prevalence of MetS and its components among obese children. **Methods:** A retrospective observational study was conducted on 800 obese patients aged 0–18 years, admitted to the “Louis Țurcanu” Children’s Clinical and Emergency Hospital in Timișoara, Romania, from 1 January 2013 to 31 December 2023. Patients were divided according to gestational age: small for gestational age (SGA), appropriate for gestational age (AGA), and large for gestational age (LGA). **Results:** Type 2 diabetes (18.2%), hypercholesterolemia (24.6%), IR (41.3%), and MetS (39.2%) were more prevalent among oSGA patients included in the study. These patients were breastfed for longer periods but weaned at a younger age. oLGA patients had the highest BMI values (28.4 ± 4.2) and, in this study group, hypertriglyceridemia (29.4%), arterial hypertension (26.8%), and lower HDL-C (41.7 ± 6.3 mg/dL) were more prevalent. The incidence of MetS increased with age (12.6 ± 3.1 years). Among these patients, IR (52.3%) was more prevalent. The introduction of flour-based energy-dense foods before six months was more frequent in MetS patients, but not statistically significant. Logistic regression showed oSGA patients had a 4.49-fold higher MetS risk (*p* < 0.001). Older age at diagnosis increased the risk of developing MetS by 37%, a diagnosis of impaired glucose tolerance by 19-fold, and a family history of diabetes by 2.7-fold. ROC analysis showed strong predictability (AUC = 0.905, sensitivity = 82%, specificity = 88%). **Conclusions:** Obese children born SGA had a higher risk for developing MetS. The incidence of MetS and its components increases with age among obese patients. Monitoring growth patterns and dietary habits in early life is paramount to mitigate future metabolic complications

## 1. Introduction

Perceived as a chronic disease with multiple complications from the onset in childhood and with major implications in adulthood, childhood obesity represents one of the greatest public health challenges of the early 21st century, especially given the dramatic increase in its prevalence in recent decades. In Romania, a study conducted by the National Institute of Public Health in 2020, according to WHO methodology and the COSI protocol (European Childhood Obesity Surveillance Initiative), shows that “3 out of 10 children aged 7–9 years are overweight or suffer from obesity” [1]. Additionally, according to a press release published by the National Institute of Public Health in March 2024, on World Obesity Day, “The incidence of obesity in children in Romania showed a slight increase until 2020 when it reached 165.94 per 100,000 inhabitants. It then increased significantly to 196.14 per 100,000 inhabitants in 2021, and 173.02 per 100,000 inhabitants in 2022” [2]. The magnitude of the obesity phenomenon is largely explained by a favorable bio-socio-economic framework, where biological predisposition, socio-economic factors, and environmental influences interact, leading to its development and perpetuation across generations.

Children who are overweight or obese face an elevated risk of developing a wide range of health conditions traditionally associated with adulthood, including type 2 diabetes, cardiovascular disorders, and metabolic syndrome [3,4]. Excessive adiposity exerts systemic effects, compromising the function of nearly all organs and physiological systems. Consequently, individuals with obesity are predisposed to a heightened incidence of gastrointestinal and hepatic diseases (e.g., non-alcoholic fatty liver disease, gastroesophageal reflux disease), respiratory conditions (e.g., obstructive sleep apnea, asthma), musculoskeletal and joint disorders (e.g., osteoarthritis), cardiovascular pathologies (e.g., cardiomyopathies, cerebrovascular accidents), and even certain malignancies beginning in childhood [3,5,6,7,8,9,10,11,12,13,14,15,16,17]. These comorbidities often persist and progressively deteriorate into adulthood, compounding the burden of disease [18].

Given the substantial long-term implications, it is imperative to investigate and elucidate the underlying mechanisms of pediatric obesity and the multifactorial contributors to its initiation and exacerbation. Such insights are critical for devising and implementing effective prevention and intervention strategies to mitigate this complex health challenge.

Although there is a genetic predisposition to obesity and the condition is influenced by a multitude of factors, lifestyle choices, high-calorie diets, and physical inactivity cannot be overlooked [19]. The roots of childhood obesity often extend to the very beginning of life, encompassing both the prenatal and postnatal periods [4,20]. Early risk factors for childhood obesity include birth-related characteristics and feeding practices during infancy and early childhood [21,22,23].

Birth weight (BW) and gestational age (GA) are essential tools for assessing neonatal health in epidemiological research and clinical practice [24]. According to some studies, infants born small for gestational age (SGA) who experienced rapid weight recovery in early postnatal life were found to have an increased long-term risk of obesity, insulin resistance (IR), and other components of metabolic syndrome (MetS) (impaired glucose tolerance, type 2 diabetes, hypertension, dyslipidemia, and cardiovascular diseases) [25,26]. On the other hand, studies also demonstrated that when assessing long-term health risks associated with infants born large for gestational age (LGA), it is important to consider the phenotype. LGA based on length is more likely genetically determined than nutritionally influenced and therefore is not associated with increased long-term risk of obesity. In contrast, LGA based on weight is a significant risk factor for obesity in adulthood [4,27].

In addition to birth characteristics, early feeding practices play a pivotal role in shaping long-term health outcomes. The way an infant is fed—whether through breastfeeding or formula feeding—the timing and nature of weaning, the age at which solid foods are introduced, and the types of foods provided, all influence the development of taste preferences, appetite regulation, and metabolism.

Recent studies have demonstrated that breastfeeding has a protective effect against developing early-onset obesity and metabolic syndrome through its impact on anthropometric indicators [28]. Conversely, formula feeding was linked to excessive weight gain and subsequent metabolic disruption [29]. It is hypothesized that bottle feeding does not allow a precise response to fullness or satiation cues, often leading to daily caloric needs being exceeded through increased feeding frequency and quantity [30].

Therefore, by understanding birth characteristics, feeding practices, and the dietary behaviors of obese children, valuable insights into the etiology and potential contributing factors to the onset and progression of childhood obesity can be provided.

This descriptive study aimed to investigate the relationship between birth characteristics—specifically birth weight and gestational age—and weaning practices, including weaning age, and feeding method (breastfeeding or formula feeding), on one hand, and body composition and metabolic effects, on the other hand, in a sample of obese children.

## 2. Materials and Methods

### 2.1. Definitions

SGA was characterized by a BW and/or length less than −2.0 standard deviations (SD) and LGA as a BW and/or length greater than +2.0 SD. Obesity was classified based on age-specific Body Mass Index (BMI) reference guidelines from the 2000 Centers for Disease Control and Prevention (CDC) Child Growth Charts [31], as BMI values above the 95th percentile.

MetS was defined as obesity along with at least two other components from the following: arterial hypertension, systolic blood pressure greater than the 95th percentile for age; disrupted glucose regulation, indicated by insulin levels over 15 mU/L or fasting blood glucose values exceeding 6.11 mmol/L; dyslipidemia, characterized by triglycerides levels of more than 1.69 mmol/L, high-density lipoprotein (HDL) cholesterol levels under 0.91 mmol/L, or total cholesterol levels over the 95th percentile. IR was diagnosed when the Homeostasis Model Assessment for Insulin Resistance (HOMA-IR) index exceeded the 95th percentile for age [32].

Further detailed definitions are provided in a previous study conducted by our research group [4].

Weaning was defined as the introduction of complementary foods (CF) after 6 months of age, following global guidelines from the World Health Organization (WHO), and early weaning as the introduction of CF before 6 months of age [33].

### 2.2. Study Design

The retrospective observational study included 800 patients aged 0–18 diagnosed with obesity, admitted to the Department of Pediatric Endocrinology, Diabetes, and Metabolic Diseases at “Louis Țurcanu” Children’s Clinical and Emergency Hospital in Timisoara, Romania, from 1 January 2013 to 31 December 2023. Anonymized patient data were obtained from the electronic register and patient files.

The study was approved by the ethics committee of “Louis Țurcanu” Children’s Clinical and Emergency Hospital in Timisoara, with no requirement for individual consent. Anonymized patient data were collected and analyzed retrospectively without the need for further intervention.

Patients were excluded from the study based on the following criteria: endocrine and/or genetic causes of SGA/LGA, pre- and post-maturity; monogenic, endocrine etiology of obesity (e.g., hypothyroidism, Cushing’s syndrome), obesity related to genetic syndromes (e.g., Prader–Willi syndrome), genetic causes of metabolic disturbances (e.g., Familial Hypercholesterolemia, Maturity-Onset Diabetes of the Young (MODY), etc.).

Patients were divided into groups according to anthropometric parameters concerning gestational age. Three main study groups were as follows: 67 obese SGA (oSGA), 598 obese AGA (oAGA), and 135 obese LGA (oLGA) subjects.

### 2.3. Measurements and Analytical Determinations

Obesity was diagnosed based on clinical assessment and anthropometric measurements, using weight (W) and height (H), to calculate the body mass index (BMI). Children under 1 year of age were measured using infant scales and meters. The Kern Baby Scale MBC 20K10EM (KERN and SOHN GmbH, Balingen, Germany), equipped with a Mechanical Height Rod MBC-A01, was used for infant measurements. Older children were evaluated using standing scales and stadiometers. Weight was measured in standing children using the KERN MPE 250K100HM Floor Scale (KERN and SOHN GmbH, Balingen, Germany). Height was measured using the Harpenden Wall-Mounted Stadiometer with a High-Speed Counter (Holtain Ltd., Felin-y-Gigfran, Crosswell, Pembrokeshire Wales, UK). BMI was calculated by dividing weight (W, in kilograms) by the square of height (H, in meters). BMI was interpreted using age- and sex-specific BMI charts [33].

Birth weight (BW) was classified into three categories as follows:

- Small for gestational age (SGA);
- Appropriate for gestational age (AGA);
- Large for gestational age (LGA).

These were based on the standard deviation (SD) criteria recorded in the patient files.

Another measured parameter was blood pressure (BP), determined by using a calibrated sphygmomanometer on two separate visits in a relaxed environment. Blood pressure was measured using either the Precisa^®^ N Sphygmomanometer or the Babyphon^®^ Sphygmomanometer (Riester, Germany), selected based on the participant’s age and arm circumference. Hypertension (HTN) was defined as systolic BP ≥ 95th percentile for the child’s age and sex [34].

The clinical evaluation of the patients, including anthropometric and blood pressure measurements, was conducted by trained personnel, specifically resident doctors under the supervision of senior physicians.

Venous blood samples were analyzed to determine glucose, insulin, and lipid profile parameters (total cholesterol, LDL, HDL, and triglycerides). These were collected after an 8 h fast and measured within 12 h. Glucose levels and lipid profiles were assessed using colorimetric enzymatic spectrophotometry with the Cobas Integra 400 Plus^®^ analyzer (Roche Diagnostics, Rotkreuz, Switzerland). Fasting insulin levels were determined by electrochemiluminescence with the Cobas e411^®^ analyzer (Roche Diagnostics). All blood analyses were performed in the certified laboratory of “Louis Țurcanu” Children’s Clinical Emergency Hospital.

The oral glucose tolerance test (OGTT) was performed according to ISPAD guidelines by measuring fasting glucose levels and glucose levels two hours after administering an oral glucose dose of 1.75 g per kilogram of body weight, up to a maximum of 75 g [35].

Blood samples were obtained by trained nurses using standard venipuncture techniques under aseptic conditions.

### 2.4. Statistical Analysis

Continuous variables were assessed for normality using the Shapiro–Wilk test. None of the variables followed a normal distribution; therefore, they were expressed as the median and interquartile range (IQR). Categorical variables were presented as numbers and percentages. For statistical analysis, the Mann–Whitney test was used to compare two continuous variables, while the Kruskal–Wallis test was applied when comparing three or more. For categorical variables, the Chi-square test was used. To evaluate the factors influencing the occurrence of metabolic syndrome, we performed a multivariable logistic regression analysis, with metabolic syndrome as the dependent variable. A backward selection method was applied, where variables were included in the model if *p* < 0.05 and excluded if *p* ≥ 0.1. The performance of the logistic regression model was assessed using a receiver operating characteristic (ROC) curve. A *p*-value of less than 0.05 was considered statistically significant. All data analyses were conducted using MedCalc^®^ Statistical Software version 23.1.1 (MedCalc Software Ltd., Ostend, Belgium; https://www.medcalc.org, accessed on 12 January 2025).

## 3. Results

All patients included in this study were evaluated, with a focus on personal history (emphasizing early feeding habits), clinical and biological characteristics as well as family history related to MetS and its components, in relation to BW and MetS.

**Figure 1:**
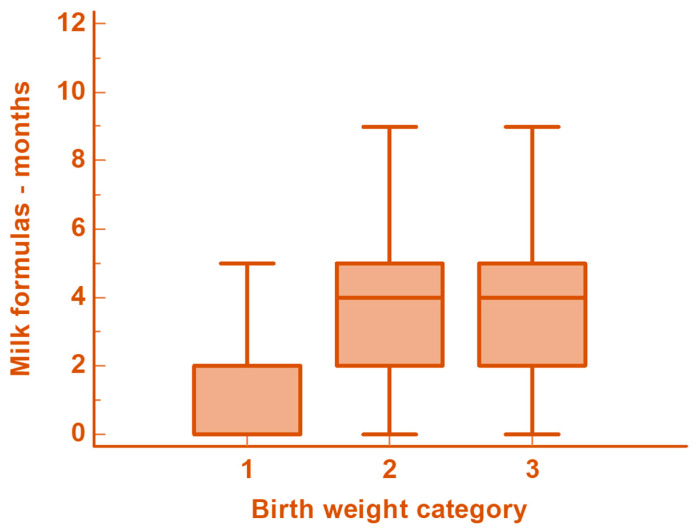
Duration of exclusive milk feedings according to BW for GA categories. 1—SGA, 2—AGA, 3—LGA.

These characteristics are summarized in Table 1, Table 2, Table 3 and Table 4 and Figure 1 Statistical analysis was performed using the Chi-square test for categorical variables and the Kruskal–Wallis and Mann–Whitney test for continuous variables to assess differences across groups.

As shown in Table 1 and Table 2, the median age was comparable across the groups. oSGA patients exhibited a lower incidence of the male sex and younger maternal age. They also showed higher incidences of type 2 diabetes, hypercholesterolemia, insulin resistance, and MetS; regarding dietary habits, they were breastfed over longer periods compared to the other two groups but were weaned earlier. In addition, the oSGA patients received formulas for shorter periods, Figure 1.

As expected, oLGA patients had the highest BMI values, as well as a higher incidence of hypertriglyceridemia and arterial hypertension, following oSGA patients closely in terms of insulin resistance, and lower HDL-c levels compared to the other groups. Arterial hypertension was detected at a higher percentage in this group but without statistical significance.

In terms of family history, oSGA children had the highest incidence of diabetes, while obesity was more prevalent among oAGA children. Arterial hypertension was most common in the oSGA and oAGA groups, with similar incidences between them.

As observed in Table 3 and Table 4, patients with metabolic syndrome were older and presented higher incidences of insulin resistance and, as expected, of MetS components. They presented lower gestational age, higher weight, and height and higher BMI at evaluation.

No differences were observed between the two groups regarding early feeding habits, including breastfeeding and formula feeding. However, as shown in Table 3, a significant difference was noted in the type of complementary foods introduced. Patients with MetS were more likely to have been introduced to flour-based and energy-dense foods before five months of age, but without reaching the statistical threshold.

To evaluate the early factors that might influence the occurrence of metabolic syndrome, an unadjusted analysis was carried out, and the results, as shown in Table 5, are expressed as odds ratio (OR) and 95%CI (confidence interval).

In this analysis, only patients from the oSGA group presented a higher risk of developing metabolic syndrome. Breastfeeding duration, milk formula duration, and diversification age were evaluated using the logistic regression method with metabolic syndrome as the dependent variable and the aforementioned variables as independent ones. None of them influenced the occurrence of metabolic syndrome.

In order to assess if the oSGA remains an independent predictor for metabolic syndrome development, a logistic regression was performed with metabolic syndrome as the dependent variable and birth weight category, age at diagnosis, glucose tolerance reduction, family history of diabetes, flour-based food before 6 months, BMI, family history of arterial hypertension, and family history of obesity as independent ones, and the results are presented in Table 6. The model proved to be good, with Nagelkerke R square = 0.41. The model excluded the missing variables from the table.

The patients with oSGA had a 4.49 higher risk of developing metabolic syndrome compared with the oAGA ones, each increase in age of diagnosis increased the risk by 37%, the presence of reduced glucose tolerance increased by almost 19 times the risk, and the family history of diabetes by 2.7 times.

A ROC curve analysis was generated to examine the performance of the logistic regression model.

**Figure 2:**
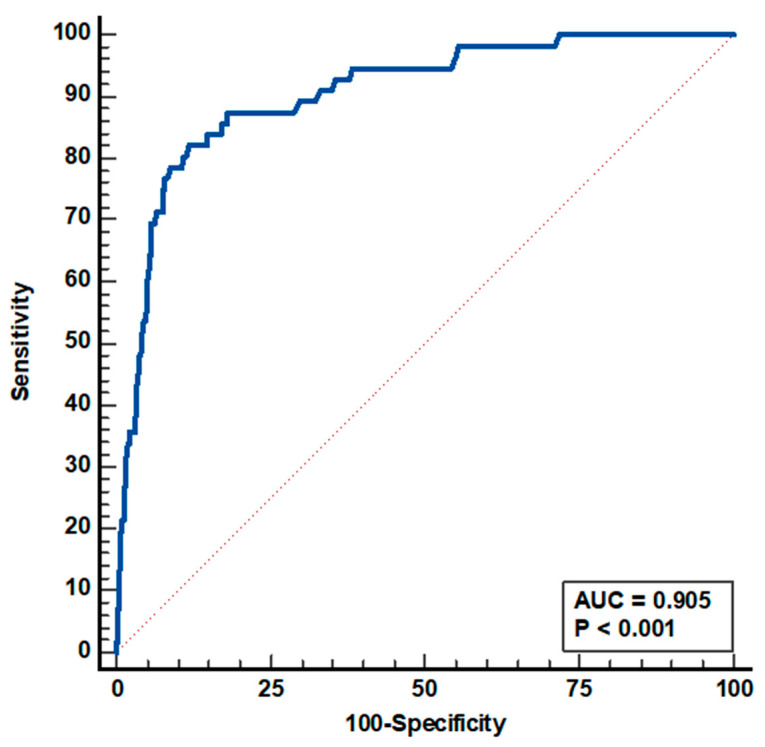
ROC curve analysis of the logistic regression model.

As shown in Figure 2, the ROC curve analysis proved to be excellent, with an AUC of 0.905 (standard error = 0.021), sensitivity of 82%, and specificity of 88%.

## 4. Discussion

Our presented descriptive study analyzed the association between birth characteristics, especially BW and GA, weaning practices, and metabolic risk. Additionally, birth metrics, feeding methods, age of weaning, and timing of CF introduction were considered in relation to body composition and metabolic effects, as well as in a sample of obese children. Metabolic outcomes, dietary habits, and family history showed significant differences among obese children born SGA, LGA, and AGA, respectively.

Children born SGA who later develop obesity are at an increased risk of developing IR and MetS or at least some of its components such as type 2 diabetes and hypercholesterolemia. Specifically, oSGA patients had a 4.49 times higher risk of developing MetS compared to the AGA group. This appears to be due to a combination of rapid catch-up growth, insulin resistance, and dysregulated glucose and lipid metabolism. Catch-up growth promotes visceral fat accumulation, increasing the probability of hyperinsulinemia with insulin resistance, which are closely linked to impaired glucose metabolism and type 2 diabetes. Impaired glucose tolerance was the strongest predictor of MetS, increasing the risk by nearly 19 times [34,35,36,37,38,39,40].

A 2.7-fold increased risk of developing MetS was noted among patients with a family history of diabetes, underlining the importance of genetic predisposition to the onset of these metabolic disorders. The data suggests that both genetic factors and those acquired in the postnatal period, including growth patterns and eating habits, contribute to the risk of MetS, especially in children born SGA [34,41,42,43,44,45,46,47].

oSGA patients were breastfed longer, but were weaned earlier than the other groups and, consequently, received exclusive milk feedings (breast milk and/or formula) for a shorter period compared to the other investigated groups. Although studies have shown that a longer duration of breastfeeding may reduce the long-term risk of metabolic diseases, including type 2 diabetes [48,49,50,51], early weaning, the reduced overall duration of breastfeeding and early weaning could indicate early exposure to solid foods, thus influencing later metabolic risk [52,53]. The very early weaning observed in the oSGA group (at 3 months of age) may have several influencing factors. One possible explanation would be the parental concern about catch-up growth, that ultimately led to the early introduction of CF in an attempt to accelerate weight gain. Socioeconomic factors and maternal perceptions of milk insufficiency also play a significant role, as parents of SGA infants may be more inclined to introduce formula or solid foods earlier to ensure adequate nutrition [54,55].

As expected, oLGA patients had the highest BMI values, along with a higher prevalence of hypertriglyceridemia, arterial hypertension, and lower HDL-c levels. These findings align with existing data in the literature, as it is known that LGA is associated with an increased metabolic risk especially when linked to obesity [56,57,58]. Additionally, oLGA patients showed a high incidence of IR, second only to oSGA patients, as fetal overfeeding has been shown to predispose individuals to insulin resistance later in life [59,60].

Analysis of family disease history revealed diabetes to be the most common among oSGA children, suggesting a genetic predisposition and early metabolic programming. However, the higher prevalence of obesity among oAGA children suggests that, while genetics and birth weight are important factors in metabolic risk, postnatal nutrition, growth patterns, and environmental factors also play a significant role [51,61,62].

In obese patients diagnosed with MetS that are included in this study, the incidence of IR, MetS, and its components increased with age [26]. However, no significant differences were observed between obese children with MetS and without MetS, in terms of eating habits in the first months of life, including breastfeeding and formula feeding. The only difference noticed was regarding the timing of CF introduction. Thus, patients with MetS were more likely to be introduced to flour-based and energy-dense foods before the age of six months. However, this difference is not significant from a statistical point of view. This observation is consistent with what is mentioned in the literature, namely that the early introduction of foods with a high caloric load could influence long-term metabolic risk, although the evidence is not yet definitive [51,62,63,64,65,66,67].

Looking at factors that could influence the appearance of MetS, it was observed that only patients in the oSGA group presented a statistically significant higher risk of developing MetS. And this observation is consistent with the findings of other researchers that children born SGA who later develop obesity are prone to insulin resistance and other metabolic complications [26,37,41].

Next, it was determined that oSGA remains a determinant risk factor for the development of MetS in obese children.

## 5. Limitations of the Study

As a retrospective observational study, the data collection relied on patients’ medical records, which may introduce selection and classification biases. Although the large number of patients is advantageous, missing or incomplete data limited the statistical analysis of important correlations, such as the direct link between low and high birth weight for gestational age and the prevalence of metabolic syndrome (MetS). Regarding the family history of the patients, it should be noted that it was not always completely evaluated in the medical records. Although, in this case, there was some incomplete data, the lack of information regarding family body shape, dietary habits, and illnesses could affect, to some extent, the analysis performed. A rather large shortcoming was the fact that there was a rather large difference between the number of patients in the SGA and LGA groups compared to the number of patients in the AGA control group. Another study limitation was the involvement of various medical staff in performing clinical measurements (anthropometric and blood pressure assessments) and blood sample collection, which, despite their training, may introduce inter-observer variability. In addition, it is possible that certain risk factors were overrepresented in the hospitalized patients analyzed compared to the general population. The absence of a lean control group limits the ability to compare findings across different weight categories, potentially affecting the generalizability of the results. Despite these limitations, the substantial sample size and the robustness of the available data enhance the relevance of our study’s conclusions.

## 6. Conclusions

Obese children born SGA have a significantly higher risk of developing MetS compared to both obese children born LGA and AGA. The incidence of MetS and its components increased with age, suggesting that the cumulative effects of these mechanisms exacerbate the metabolic risk over time.

Both personal and family history of impaired glucose metabolism and diabetes, respectively, had a significant influence on the development of MetS.

Exclusive milk feeding, whether breastfeeding or formula feeding, did not significantly influence the incidence of MetS in obese children included in this study. However, early introduction of flour-based and energy-dense CF before six months of age was more common among children with MetS, indicating that early dietary practices may influence long-term metabolic risk.

Therefore, monitoring growth patterns and dietary habits in early life is crucial to mitigate future metabolic complications.

## Figures and Tables

**Table 1:** Assessment of personal and family history, along with anthropometric parameters, stratified by BW.

*Variable*	*oSGA*	*oAGA*	*oLGA*	*Total*	*p Value*
	*No. 67*	*No. 598*	*No. 135*	*No. 800*	
***Sex: males***	30 (44.8%)	340 (56.9%)	92 (68.7%)	462 (57.8%)	0.0008
***Mother’s age at birth (years)*** ***M + IQR***	32.5 (30–38)	34 (31–39)	38 (36–39.5)	35 (31–39)	0.0292 oLGA vs. oAGA and oSGA
***Gestational age (weeks)*** ***M + IQR***	38 (38–38)	38 (38–40)	40 (38–40)	39 (38–40)	<0.0001 oSGA vs. oAGA and oLGA, oAGA vs. oLGA
***Length at birth (cm)*** ***M + IQR***	55 (50.25–59)	54 (51–57)	53 (48–56.5)	54 (50.75–57)	0.454
***Weight at birth (g)*** ***M + IQR***	2200 (2010–2357.5)	3250 (3000–3600)	4300 (4000–5800)	3300 (2980–3750)	<0.0001 between all three groups
***Breastfeeding (months)*** ***M + IQR***	2.5 (1–3.5)	1.125 (0–3)	1 (0–3)	1.5 (0–3)	0.0043 oSGA vs. oAGA and oLGA
***Formula (months)*** ***M + IQR***	2 (1–3)	4 (3–5)	4 (2–5)	4 (2–5)	<0.0001 oSGA vs. oAGA and oLGA
***Weaning age (months)*** ***M + IQR***	3 (3–4)	5 (5–6)	5 (5–6)	5 (4–6)	<0.0001 oSGA vs. oAGA and oLGA
***Flour-based/energy-dense foods < 6 months***	2 (3%)	25 (4.2%)	0 (0%)	27 (3%)	0.005
***Age at evaluation (years)*** ***M + IQR***	11.9 (9.77–14.35)	12.11 (9.4–14.9)	12.1 (9.72–14.8)	12.1 (9.5–14.8)	0.814
***Weight at evaluation (kg)*** ***M + IQR***	60 (42.92–69.62)	64 (46–85)	70 (53–86)	64 (47–84.35)	0.0138 oSGA vs. oAGA and oLGA
***Height at evaluation (cm)*** ***M + IQR***	147 (132.25–156)	153 (140–163)	155 (145–163)	153 (140.5–163)	0.0154 oSGA vs. oAGA and oLGA
***BMI at evaluation (kg/m^2^)*** ***M + IQR***	26.77 (23.54–30.68)	27.23 (23.61–32.87)	28.72 (25.03–33.38)	27.66 (23.8–32.82)	0.0306 oLGA vs. oAGA and oSGA
***Family History***					
***Diabetes***	12 (17.9%)	65 (10.9%)	13 (5.3%)	90 (9.9%)	0.0034
***Obesity***	3 (4.5%)	62 (10.4%)	6 (2.4%)	71 (7.8%)	0.0003
***Arterial Hypertension***	8 (11.9%)	72 (12%)	8 (3.3%)	88 (9.7%)	0.0004

Legend: M + IQR, median and interquartile range; oSGA, obese small birth weight for gestational age; oAGA, obese appropriate birth weight for gestational age; oLGA, obese large birth weight for gestational age; BMI, body mass index.

**Table 2:** Evaluation of the MetS and components, stratified by BW.

*Variable*	*oSGA*	*oAGA*	*oLGA*	*Total*	*p Value*
	*No. 67*	*No. 598*	*No. 135*	*No. 800*	
***Impaired glucose metabolism***					
***Type 2 diabetes***	6 (9%)	5 (0.8%)	0 (0%)	11 (1.4%)	<0.0001
***Impaired glucose tolerance***	13 (19.4%)	120 (20.1%)	36 (26.7%)	169 (21.1%)	0.222
***Insulin resistance***	25 (37.3%)	164 (27.4%)	49 (36.3%)	238 (29.8%)	0.0462
***Impaired lipid metabolism***					
***Hypercholesterolemia***	11 (16.4%)	35 (5.9%)	21 (15.6%)	67 (8.4%)	0.0001
***Hypertriglyceridemia***	11 (16.4%)	56 (9.4%)	28 (20.7%)	95 (11.9%)	0.0005
***Arterial hypertension***	7 (10.4%)	46 (7.7%)	17 (12.6%)	70 (8.8%)	0.167
***Metabolic Syndrome***	9 (13.4%)	32 (5.4%)	15 (6.1%)	56 (6.1%)	0.033

Legend: M + IQR, median and interquartile range; oSGA, obese small birthweight for gestational age; oAGA, obese appropriate birthweight for gestational age; oLGA, obese large birthweight for gestational age; HDL, high-density lipoproteins.

**Table 3:** History and clinical assessment of the patients stratified by the presence of MetS.

*Variable*	*MetS*	*No MetS*	*p-Value*
	*No. 56*	*No. 744*	
***Sex: males***	35 (62.5%)	427 (57.5%)	0.462
***Mother’s age at birth (years)*** ***M + IQR***	37 (33.5–47)	35 (31–38.5)	0.0644
***Gestational age (weeks)*** ***M + IQR***	38 (38–39)	39 (38–40)	0.0006
***Length at birth (cm)*** ***M + IQR***	56 (51.25–58)	54 (50–57)	0.335
***Weight at birth (g)*** ***M + IQR***	3500 (2800–3900)	3300 (3000–3750)	0.792
***Breastfeeding (months)*** ***M + IQR***	1.5 (1–3)	1.5 (0–3)	0.4698
***Formula (months)*** ***M + IQR***	3.75 (2–5)	4 (2–5)	0.117
***Weaning age (months)*** ***M + IQR***	5 (4–6)	5 (4–6)	0.248
***Flour-based/energy-dense foods < 6 months***	4 (7.1%)	23 (2.7%)	0.057
***Age at evaluation (years)*** ***M + IQR***	15.55 (14.1–16.75)	12 (9.3–14.2)	<0.0001
***Weight at evaluation (kg)*** ***M + IQR***	87 (72.5–97.5)	62 (45–81.25)	<0.0001
***Height at evaluation (cm)*** ***M + IQR***	162.5 (156–167)	152 (138.75–162.75)	<0.0001
***BMI at evaluation*** ***(kg/m^2^)*** ***M + IQR***	32.31 (29.75–35.74)	27.18 (23.61–32.52)	<0.0001
***Family History***			
***Diabetes***	9 (16.1%)	81 (9.5%)	0.1
***Obesity***	6 (10.7%)	65 (7.6%)	0.4
***Arterial Hypertension***	7 (12.5%)	81 (9.5%)	0.45

Legend: M + IQR, median and interquartile range; oSGA, obese small birth weight for gestational age; oAGA, obese appropriate birth weight for gestational age; oLGA, obese large birth weight for gestational age; BMI, body mass index.

**Table 4:** Diagnostic and biochemical evaluation of patients stratified by the presence of MetS and components.

*Variable*	*MetS*	*No MetS*	*p Value*
	*No. 56*	*No. 744*	
***Impaired glucose metabolism***			
***Type 2 diabetes***	4 (7.1%)	7 (0.9%)	0.0001
***Impaired glucose tolerance***	46 (82.1%)	123 (16.5%)	<0.0001
***Insulin resistance***	41 (73.2%)	197 (26.5%)	<0.0001
***OGTT: Fasting*** ***glucose (mmol/L)*** ***M + IQR***	4.86 (4.23–5.42)	4.79 (4.33–5.34)	0.986
***OGTT:*** ***2 h-glucose (mmol/L)*** ***M + IQR***	6.82 (6.42–7.68)	6.77 (6.28–7.39)	0.648
***Insulinemia (μUI/mL)***	10.2 (6.74–27.07)	9.41 (6.33–16.2)	0.1
***HOMA-IR M + IQR***	2.13 (1.21–6.35)	1.78 (1.14–2.92)	0.17
***Impaired lipid metabolism***			
***Hypercholesterolemia***	16 (28.6%)	51 (6.9%)	<0.0001
***Hypertriglyceridemia***	39 (69.6%)	56 (7.5%)	<0.0001
***Lipids (g/L)*** ***M + IQR***	6.55 (6–7)	5.4 (4.8–6)	0.013
***Total cholesterol (mmol/L)*** ***M + IQR***	4.44 (4.11–4.92)	3.97 (3.53–4.66)	0.0402
***HDL Cholesterol (mmol/L)*** ***M + IQR***	1.63 (1.11–2.08)	1.69 (1.21–2.42)	0.237
***LDL-c (mmol/L)*** ***M + IQR***	1.02 (0.69–1.66)	1.33 (0.75–2.2)	0.1607
***Triglycerides (mmol/L)*** ***M + IQR***	1.04 (0.73–1.48)	0.89 (0.66–1.21)	0.0807
***Triglycerides to HDL-c ratio*** ***M + IQR***	0.7 (0.37–0.99)	0.52 (0.34–0.84)	0.077
***Arterial hypertension***	36 (64.3%)	34 (4.6%)	<0.0001

Legend: M + IQR, median and interquartile range; MetS, metabolic syndrome; OGTT, oral glucose tolerance test; HOMA-IR, homeostatic model assessment insulin resistance index; HDL, high-density lipoproteins; LDL, low-density lipoproteins.

**Table 5:** Perinatal and inherited factors influencing MetS development.

*Variable*	*Unadjusted OR and 95% CI*	*p Value*
***oSGA***	2.63 (1.22–5.63)	0.0127
***oAGA***	0.68 (0.39–1.17)	0.169
***oLGA***	0.98 (0.53–1.81)	0.968
***Diabetes family history***	1.82 (0.86–3.86)	0.113
***Obesity family history***	1.45 (0.6–3.52)	0.402
***Arterial hypertension family history***	1.36 (0.59–3.11)	0.459

Legend: OR, odds ratio; CI, confidence interval.

**Table 6:** Logistic regression of factors that influence the MetS.

*Variable*	*OR*	*95% CI*	*p Value*
***oSGA***	4.59	1.77–11.9	0.0017
***Age at diagnosis (years)***	1.37	1.21–1.54	<0.0001
***Glucose tolerance reduction (yes/no)***	18.94	8.73–41.1	0.0001
***Family history of diabetes***	2.69	1.11–6.51	0.0274
***AUC***	0.905	0.883–0.925	<0.0001

Legend: OR, odds ratio; CI, confidence interval; OSGA, small birth weight; AUC, area under the curve.

## Data Availability

The original contributions presented in this study are included in the article, and further inquiries can be directed to the corresponding author at the e-mail address chi-savu.lazar@umft.ro. The raw data supporting the conclusions of this article will be made available by the authors upon request. All of the data are presented in the current form of the manuscript.
